# Topography of respiratory tract and gut microbiota in mice with influenza A virus infection

**DOI:** 10.3389/fmicb.2023.1129690

**Published:** 2023-02-22

**Authors:** Qichao Chen, Manjiao Liu, Yanfeng Lin, Kaiying Wang, Jinhui Li, Peihan Li, Lang Yang, Leili Jia, Bei Zhang, Hao Guo, Peng Li, Hongbin Song

**Affiliations:** ^1^Academy of Military Medical Sciences, Academy of Military Sciences, Beijing, China; ^2^Chinese PLA Center for Disease Control and Prevention, Beijing, China; ^3^State Key Laboratory of Translational Medicine and Innovative Drug Development, Jiangsu Simcere Diagnostics Co., Ltd., Nanjing, Jiangsu Province, China; ^4^Nanjing Simcere Medical Laboratory Science Co., Ltd., Nanjing, Jiangsu Province, China

**Keywords:** influenza A virus infection, respiratory tract microbiota, gut microbiota, oropharynx, nasopharynx, lung

## Abstract

**Introduction:**

Influenza A virus (IAV)-induced dysbiosis may predispose to severe bacterial superinfections. Most studies have focused on the microbiota of single mucosal surfaces; consequently, the relationships between microbiota at different anatomic sites in IAV-infected mice have not been fully studied.

**Methods:**

We characterized respiratory and gut microbiota using full-length 16S rRNA gene sequencing by Nanopore sequencers and compared the nasopharyngeal, oropharyngeal, lung and gut microbiomes in healthy and IAV-infected mice.

**Results:**

The oropharyngeal, lung and gut microbiota of healthy mice were dominated by *Lactobacillus* spp., while nasopharyngeal microbiota were comprised primarily of *Streptococcus* spp. However, the oropharyngeal, nasopharyngeal, lung, and gut microbiota of IAV-infected mice were dominated by *Pseudomonas, Escherichia*, *Streptococcus*, and *Muribaculum* spp., respectively. *Lactobacillus murinus* was identified as a biomarker and was reduced at all sites in IAV-infected mice. The microbiota composition of lung was more similar to that of the nasopharynx than the oropharynx in healthy mice.

**Discussion:**

These findings suggest that the main source of lung microbiota in mice differs from that of adults. Moreover, the similarity between the nasopharyngeal and lung microbiota was increased in IAV-infected mice. We found that IAV infection reduced the similarity between the gut and oropharyngeal microbiota. *L. murinus* was identified as a biomarker of IAV infection and may be an important target for intervention in post-influenza bacterial superinfections.

## Introduction

The risk of a novel influenza A virus (IAV) pandemic looms as a major global public health threat (Iuliano et al., 2018). Secondary bacterial infection following influenza is a prevalent cause of severe pneumonia and death (McCullers, 2014). The normal host microbiome resists colonization by pathogens through niche competition and host immune regulation, and plays an important role in post-influenza bacterial superinfection (Dominguez-Bello et al., 2019).

The intestinal microbiome includes 10^14^ bacteria representing 1,000 species, which are closely related to host metabolism, immunity and mental health (Lynch and Pedersen, 2016). Patients with IAV infection exhibited significantly decreased diversity and abundance of gut microbiota, including a significant reduction in the relative abundance of *Actinomycetes* and *Firmicutes* at the phylum level and anaerobic butyrate-producing bacteria (*Racinobacteriaceae* and *Ruminococcaceae*) at the family level (Gu et al., 2020). Intestinal dysbiosis dysregulates host CD4+ and CD8+ T cell generation and antibody response, and aggravates IAV-induced lung pathology (Ichinohe et al., 2011). In addition to the gut microbiome, respiratory microbiota also plays an important role in IAV infection. Due to differences in anatomy and development, the human respiratory tract can be divided into the upper and lower respiratory tracts (URT and LRT, respectively). IAV infection can significantly alter the URT microbiome, including oropharyngeal and nasopharyngeal microbiota. For example, IAV-infected children had lower abundance of *Moraxella*, *Staphylococcus*, *Clostridium* and *Duchenne* spp. in the nasopharynx, and *Streptococcus*, *Neisseria* and *Hemophilus spp.* in the oropharynx than healthy children (Wen et al., 2018). In healthy adults, lung microbiota originates primarily from the oropharynx by direct mucosal dispersion and micro-aspiration (Bassis et al., 2015). A murine model demonstrated that IAV infection induced a long-term LRT dysbiosis that featured a clear shift from *Alphaproteobacteria* to *Gammaproteobacteria* and a significant increase in the relative abundance of *Streptococcus* and *Staphylococcus* spp. (Gu et al., 2019). Although multiple studies have characterized the changes of respiratory tract and gut microbiota after IAV infection, most have focused on specific anatomic sites. Correlations between URT, LRT and gut microbiota during IAV infection have not been studied fully. In addition, due to the read length limitation of second-generation sequencing, few species-level studies of host dysbiosis during IAV infection have been conducted.

To further understand the changes and correlation between URT, LRT and gut microbiota during IAV infection, we performed full length 16sRNA gene sequencing of nasopharyngeal, oropharyngeal, lung and gut microbiota using Nanopore sequencing technology in a murine model. We found that IAV infection altered the microbiota structures of the URT, LRT and gut. The lung microbiota was more similar to nasopharyngeal than oropharyngeal microbiota in healthy mice. However, the similarity between nasopharyngeal and lung microbiota deceased during IAV-infection. We also observed that IAV infection reduced the similarity between gut and oropharyngeal microbiota. In addition, we found representative species responses to IAV infection. For example, IAV infection decreased the relative abundance of *Lactobacillus murinus* in the respiratory tract and gut.

## Materials and methods

### Animal model and sample collection

Twenty C57BL/6 N female mice (6 weeks of age) were purchased from Beijing Vital River Laboratory Animal Technology Co., Ltd. (China) and adapted in specific pathogen-free conditions (5 mice/cage) for 1 week. Mice were randomly divided into two groups (*n* = 10): a PBS mock infected (Mock) group and an IAV-infected (IAV) group. To establish a murine model of IAV infection, mice of the IAV group were anesthetized with 0.3% pentobarbital sodium (intraperitoneal injection, 50 mg/kg) and infected intranasally with 25 μl sterile PBS containing strain A/Puerto Rico/8/34 (60 PFU). Control mice were mock infected with 25 μl sterile PBS. One nasal drip experiment for each mouse. All mice in the IAV group and Mock group were infected in the same day. All mice had access to water and food under a strict 12 h light/dark cycle. All animal procedures for animal raising and handling were approved by the Animal Care and Use Committee of Chinese PLA Center for Disease Control and Prevention. All animals were euthanized with 0.3% pentobarbital sodium (intraperitoneal injection, 150 mg/kg) on post-infection day-4. After weighing the fresh lungs, left lungs were stored at-80°C and right lungs were fixed in 4% paraformaldehyde for hematoxylin and eosin (H&E) staining. The lung index was calculated with the formula: lung index = [(lung weight/g)/(bodyweight/g)] × 100% (Gao et al., 2020). Nasopharyngeal lavage fluid (NLF) was collected as reported previously (Puchta et al., 2014). Oropharyngeal samples were collected using swabs and placed in 1 ml sterile PBS at 4°C. Bronchoalveolar lavage fluid (BALF) was collected by washing the bronchoalveolar tree three times using 1 ml sterile PBS. Fecal samples were collected and stored at-80°C for further experiments.

### Hematoxylin and eosin staining

Fresh right lung tissues were fixed in 4% paraformaldehyde and embedded in paraffin. Prepared embedded lung tissues were cut into 3–5 μm-thick sections and stained with H&E. Pathological scores were evaluated as reported previously (Renne et al., 2009).

### Nucleic acid extraction and qPCR

Bacterial genomic DNA of fresh NLF, BALF and oropharyngeal swabs was extracted using the QIAamp DNA Microbiome Kit (QIAGEN, Germany). Total RNA of fresh NLF, BALF and oropharyngeal swabs was extracted using QIAamp^®^ MinElute^®^ Virus Spin (QIAGEN, Germany). Genomic DNA and total RNA of fecal samples were extracted using AllPrep^®^ PowerFecal^®^ DNA/RNA Kit (QIAGEN, Germany). A control extraction with no sample was performed for each kit. IAV titers of NLF, BALF, oropharyngeal swab and fecal samples were assayed with Luna^®^ Universal Probe One-Step RT-qPCR Kit (NEB, United States). The following primers were used: 5’-GACCRATCCTGTCACCTCTGAC-3′ (Forward primer), 5’-GGGCATTYTGGACAAAKCGTCTACG-3′ (Reverse primer) and 5’-FAM-TGCAGTCCTCGCTCACTGGGCACG-BHQ1-3′ (Probe). Copies of IAV were calculated using the standard curve method.

### Full length 16S rRNA gene sequencing and bioinformatics pipeline

Genomic DNA of NLF, BALF, oropharyngeal swab and fecal samples was used for full length 16S rRNA gene sequencing. In addition to the samples, library preparation contained a negative control (Control extraction with no sample) and a positive control (mock community). PCR reactions were conducted using KAPA HiFi HotStart ReadyMix (KAPA Biosystems, United States) and 0.5 μM of Universal-27F (5′- TTTCTGTTGGTGCT GATATTGCAGAGTTTGAT CCTGGCTCAG-3′) and Universal-1492R (5′- ACTTGCC TGTCGCTCTATCTTCTACGACTTAACCCCAATCGC-3′). Cycling conditions were set as 95°C for 3 min; 10 cycles of 98°C for 30 s, 55°C for 1 min and 72°C for 1 min; and 72°C for 5 min. The amplification products were purified using 0.8 × AgencourtAMPure XP Beads (Beckman, United States). Cleaned DNA was barcoded and pooled using PCR Barcoding Expansion Pack 1–96 (EXP-PBC096; Oxford Nanopore Technologies, United Kingdom). A library was prepared for sequencing using Ligation Sequencing Kit (SQK-LSK109; Oxford Nanopore Technologies, United Kingdom) and sequenced on the MinION Mk1B (Oxford Nanopore Technologies, United Kingdom) with R10 flow cell (Oxford Nanopore Technologies, United Kingdom). Raw reads were base-called and demultiplexed using Guppy (V 5.0.11+) to obtain high-quality reads with min_score = 8. The filtered reads were within a size range of 1.2–1.8 kb. Read numbers and mapping rates for each sample are presented in Supplementary Table S1. Emu software was used to estimates species composition distribution based on the Emu v3.0 + database (Curry et al., 2022). Alpha diversity (Shannon and Chao1 indices) and Bray Curtis Distance were calculated by R package vegan (v2.5.7). The principal coordinate analysis (PCoA) was formed by R package ape (v5.6.2). Functional composition and KEGG pathway abundance of microbiota was predicted using PICRUST2 software (Douglas et al., 2022). LEfse analysis was used to identify biomarkers by comparing abundance between groups (Wilcoxon test *p* < 0.01 and |log10(LDA)| >3).

### Statistics

Body weight, IAV titer and histologic scoring were analyzed with unpaired t tests. Shannon index, Chao1 index and bacterial taxa abundance among groups were analyzed with the Mann–Whitney test. ***p ≤ 0.001, **p ≤ 0.01, *p ≤ 0.05.

## Results

### IAV induced severe respiratory tract infection and lung injury

**Figure 1:**
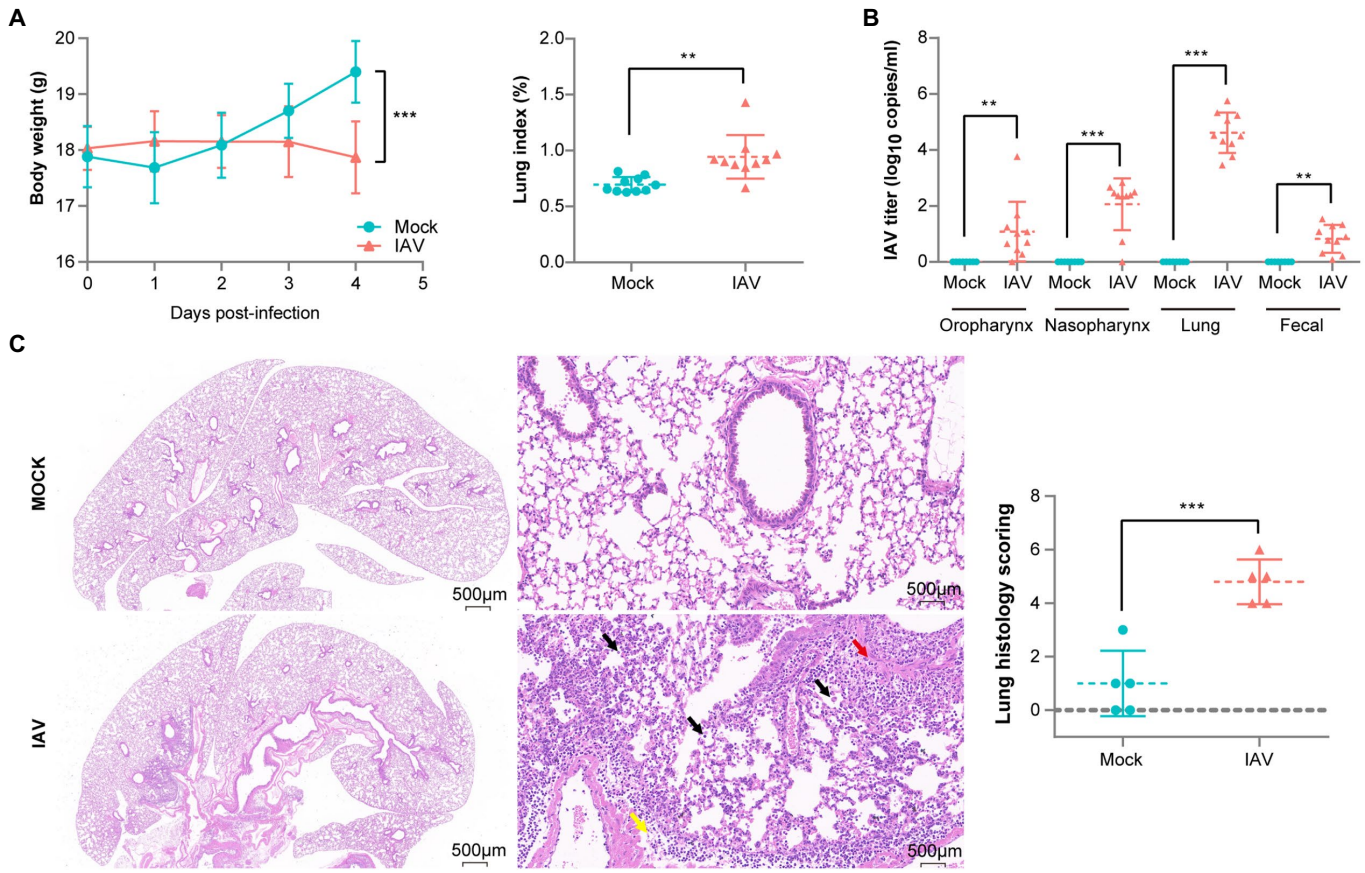
Pathological difference between mock-and IAV-infected mice. **(A)** Body weight and lung index (*n* = 10 per group). **(B)** IAV titers of oropharynx, nasopharynx, lung and fecal samples (*n* = 10 per group). **(C)** H&E staining and histologic scoring of right lungs (*n* = 5 per group). Body weight, IAV titer and histologic scoring were compared by using unpaired t tests *p < 0.05, ** *p* < 0.01, ***p < 0.001.

To validate our murine infection model, we compared body weight, IAV titer and lung pathology between mock and IAV groups. A significant increase in lung index but a significant decrease in body weight were observed in the IAV group compared to the mock group (Figure 1A). We also observed significantly increased IAV titers in the oropharynx, nasopharynx, lung and fecal samples of the IAV group and found the highest IAV titers in the lung (Figure 1B). H&E staining showed that pulmonary injury was induced by IAV infection and featured alveolar wall thickening (Yellow arrow), mononuclear cell infiltration (Black arrows) and bronchial epithelial cell injury (Red arrow). The mean histologic score of the IAV group was significantly higher than that of the mock group (Figure 1C).

### IAV infection altered the composition of respiratory tract microbial communities

**Figure 2:**
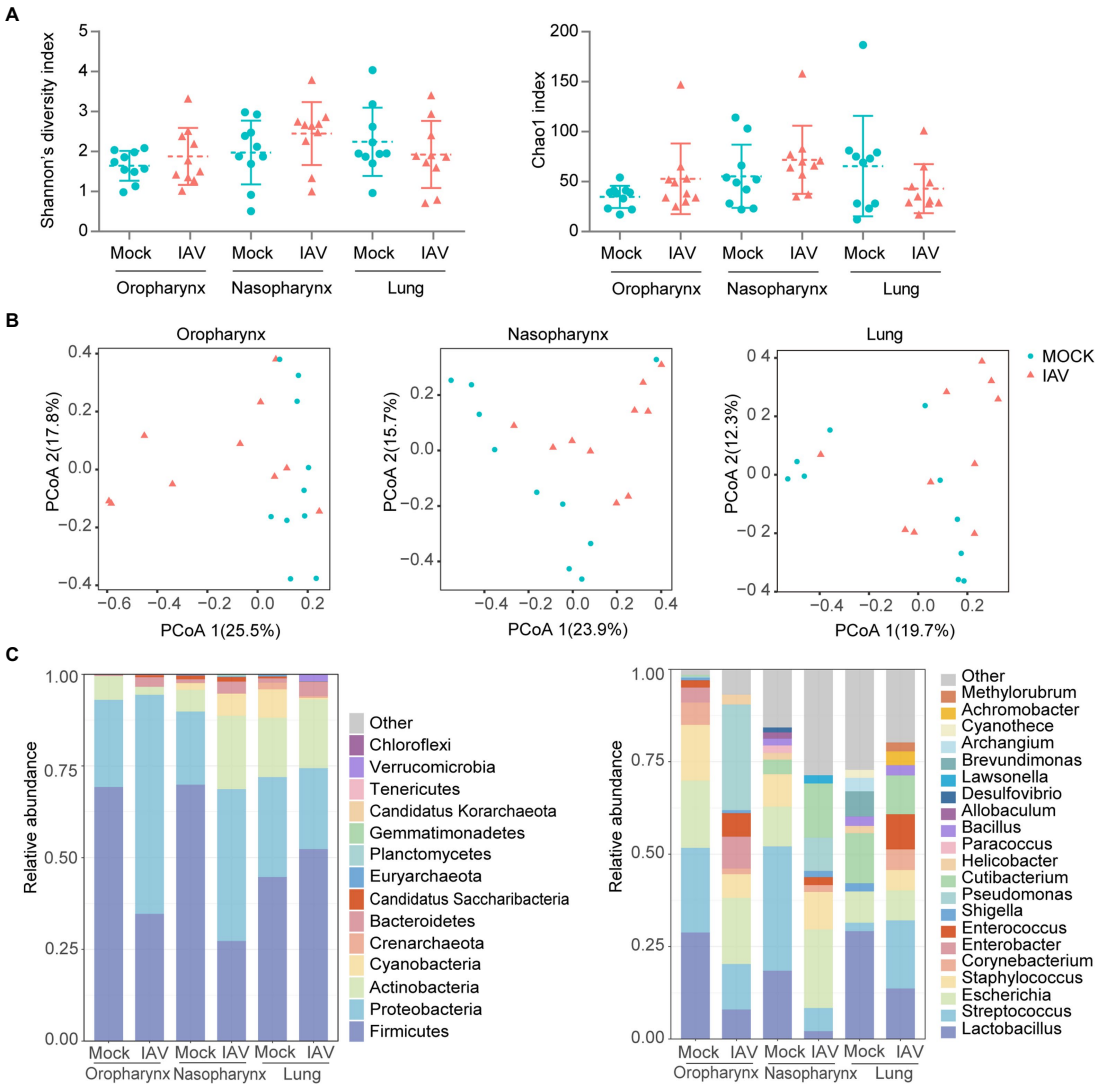
Respiratory tract dysbiosis during IAV infection. **(A)** Shannon diversity and Chao1 indices of oropharyngeal, nasopharyngeal and lung microbiota. **(B)** 2D-PCoA plots of oropharyngeal, nasopharyngeal and lung microbiota. Relative abundance of the top 10 bacteria at the phylum and genus levels **(C)**. Shannon and Chao1 indices were analyzed with Mann–Whitney test. *p < 0.05, ** *p* < 0.01, ***p < 0.001. *n* = 10 per group.

The diversity (Shannon diversity index) and richness (Chao1 index) of the oropharyngeal, nasopharyngeal and lung microbiota were similar between the two groups (Figure 2A). PCoA analysis showed clearly different oropharyngeal and nasopharyngeal microbiota in IAV group compared with the mock group; however, lung microbiota structures were not significantly different (Figure 2B; Supplementary Figures S1A,B,C).

**Figure 3:**
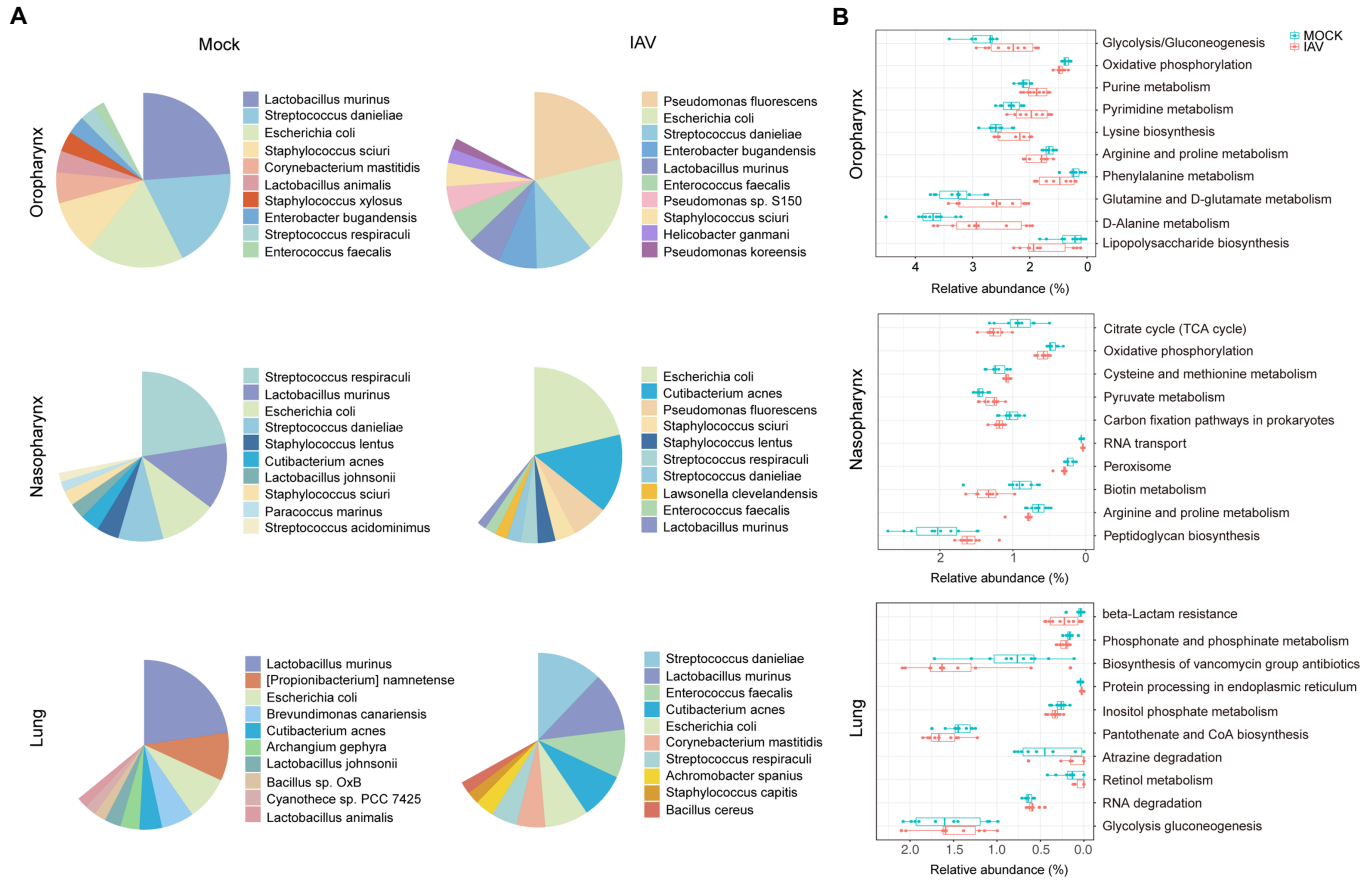
Characterization of species-level bacteria and functional composition of respiratory tract microbiota during IAV infection. **(A)** Relative abundance of the top 10 bacterial species of oropharyngeal, nasopharyngeal and lung microbiota in mock and IAV groups. **(B)** Top 10 significantly altered metabolic pathways of oropharyngeal, nasopharyngeal and lung microbiota between mock and IAV groups. Pathway abundance prediction by PICRUSt2. *n* = 10 per group. The relative abundance on the x-axis represents the ratio of the corresponding functional abundance to all predicted functional abundances.

We further examined taxonomic profiles at different classification levels. The most prominent phyla of all samples in the mock group were *Proteobacteria* and *Firmicutes*, while the IAV group had a relatively reduced abundance of *Firmicutes* but increased abundance of *Proteobacteria* in the oropharynx and nasopharynx. However, these changes were not observed in the lung. At the genus level, *Lactobacillus* was most abundant in oropharynx and lung, whereas *Streptococcus* was predominant in the nasopharynx of the mock group. However, the IAV group exhibited different dominant genera at all locations. *Pseudomonas* was dominant in the oropharynx, whereas *Escherichia* was most abundant in nasopharynx and *Streptococcus* was predominant in the lung (Figure 2C). We also investigated species-level changes of respiratory tract microbiota during IAV infection. For the mock group, *L. murinus* was predominant in oropharynx and lung, whereas *Streptococcus respiraculi* was most abundant in nasopharynx. For the IAV group, *Pseudomonas fluorescens* was predominant in oropharynx, *Escherichia coli* was predominant in nasopharynx, whereas *Streptococcus respiraculi* was most abundant in lung (Figure 3A). The metabolic function prediction of microbiota showed that IAV infection decreased D-glutamine, D-glutamate and D-Alanine metabolism in the oropharynx and reduced peptidoglycan biosynthesis in the nasopharynx, while increasing pantothenate and CoA biosynthesis in the lung (Figure 3B).

### Comparison of microbiota structures of respiratory tract sites during IAV infection

**Figure 4:**
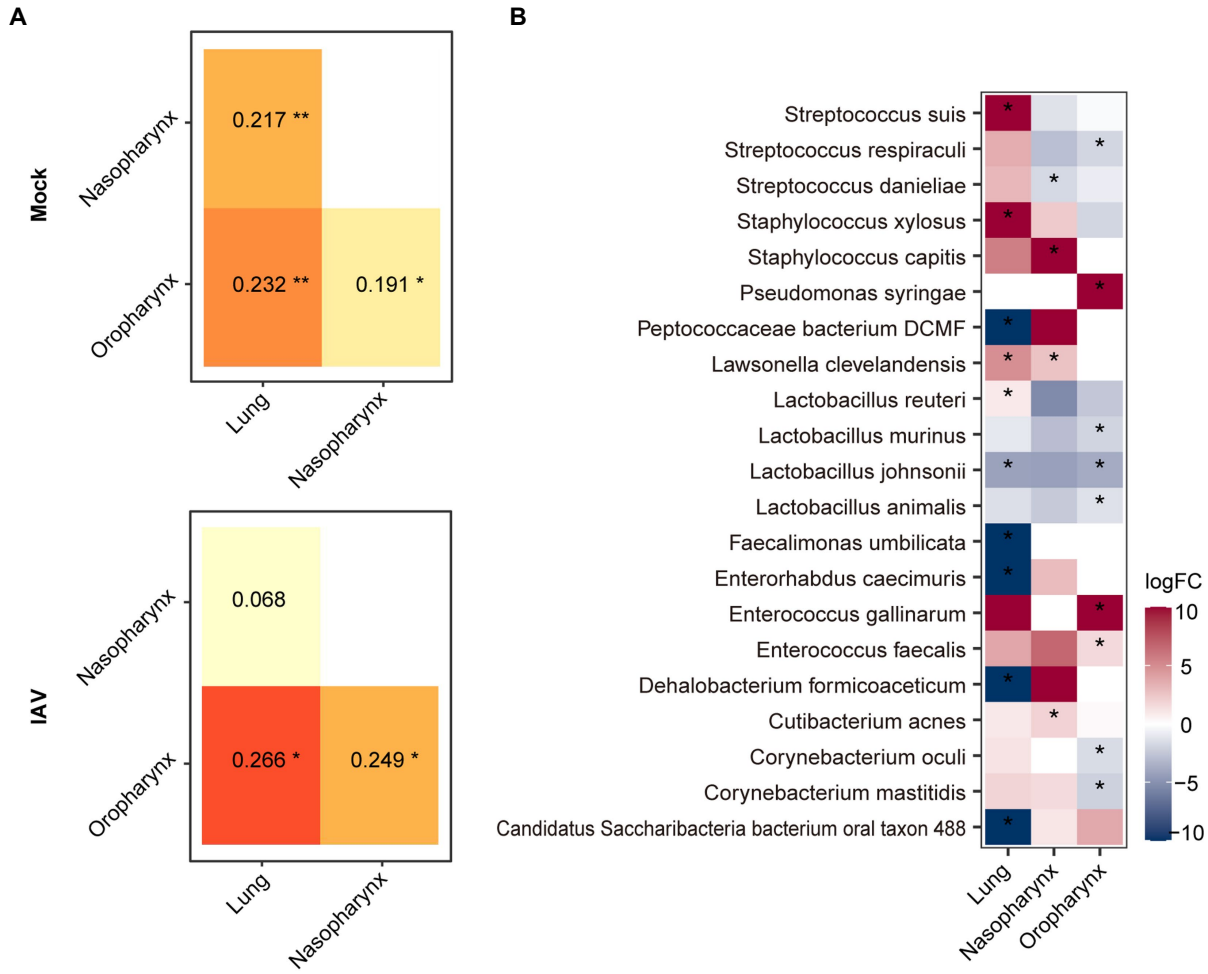
Comparison of oropharyngeal, nasopharyngeal and lung microbiota during IAV infection. **(A)** Similarity analysis of the microbiota of different locations in the IAV and mock groups. **(B)** Distribution of significantly different species between mock and IAV groups in different sampling locations. Shannon and Chao1 indices among different sampling locations were analyzed with the Mann–Whitney test. Similarity of microbiota of different locations was analyzed with ANOSIM. Bacterial taxa abundance between mock and IAV group was also analyzed with the Mann–Whitney test. ***p < 0.001, **p < 0.01, *p < 0.05. *n* = 10 per group.

We found that the lung microbiota was more similar to nasopharyngeal than oropharyngeal microbiota in the mock group. Moreover, the similarity between lung and nasopharyngeal microbiota was increased in the IAV group compared to the mock group (Figure 4A). These results suggest that IAV infection increased the influence of nasopharyngeal microbiota on lung microbiota. We screened the species with significant differences between the IAV group and the mock group by the Mann–Whitney test and showed the changes at each location on the heat map. Compared with respective locations in mock group, the oropharynx showed a significant difference of 18 species, the nasopharynx displayed a significant difference of 7 species and the lung exhibited a significant difference of 13 species. We identified some species with synchronous alterations at multiple respiratory tract locations in response to IAV infection. For example, compared with mock group, the relative abundance of *Lactobacillus animalis*, *Lactobacillus johnsonii* and *L. murinus* decreased, while the relative abundance of *Enterococcus faecalis* increased in the oropharynx, nasopharynx and lungs in IAV group. In addition, we found that some species of the URT and LRT responded differently to IAV infection. For example, the relative abundance of *S. danieliae*, *S. respiraculi and Streptococcus suis* decreased in the URT but increased in the LRT in the IAV group (Figure 4B).

### IAV infection reduced the similarity between oropharyngeal and gut microbiota

**Figure 5:**
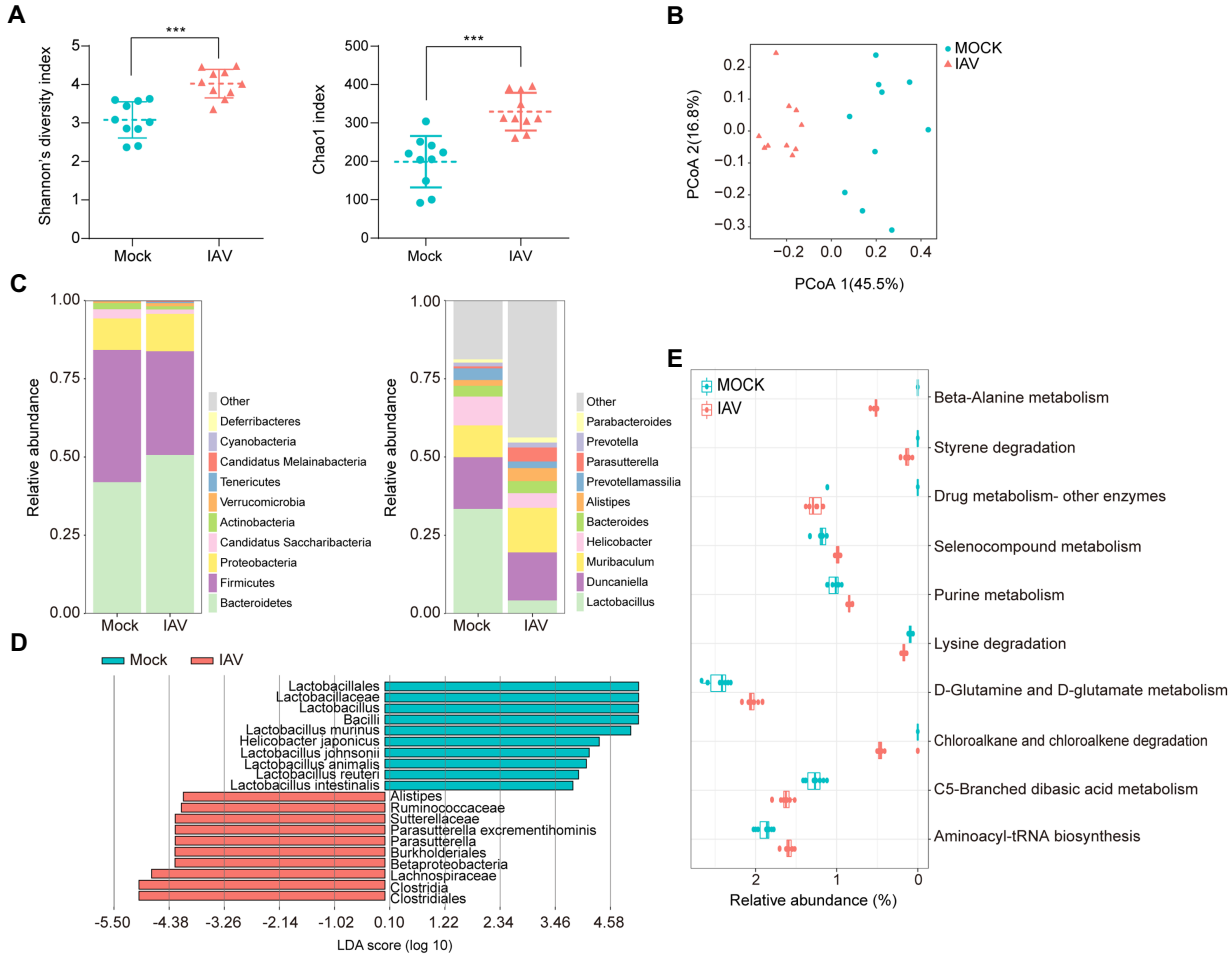
Characterization of gut microbiota and correlation between gut and oropharyngeal microbiota during IAV infection. **(A)** Shannon diversity and Chao1 indices of gut microbiota in IAV and mock groups. **(B)** 2D-PCoA plots of gut microbiota in mock and IAV groups **(C)** Relative abundance of the top 10 bacteria at the phylum and genus levels of gut microbiota in mock and IAV groups. **(D)** LEfSe analysis at the species level of gut microbiota in mock and IAV groups. **(E)** Top 10 significantly altered metabolic pathways of gut microbiota between mock and IAV groups. Pathway abundance prediction by PICRUSt2. Statistical analyses of Shannon index, Chao1 index and bacterial taxa abundance were conducted by using the Mann–Whitney test. *p < 0.05, ** *p* < 0.01, ***p < 0.001.

**Figure 6:**
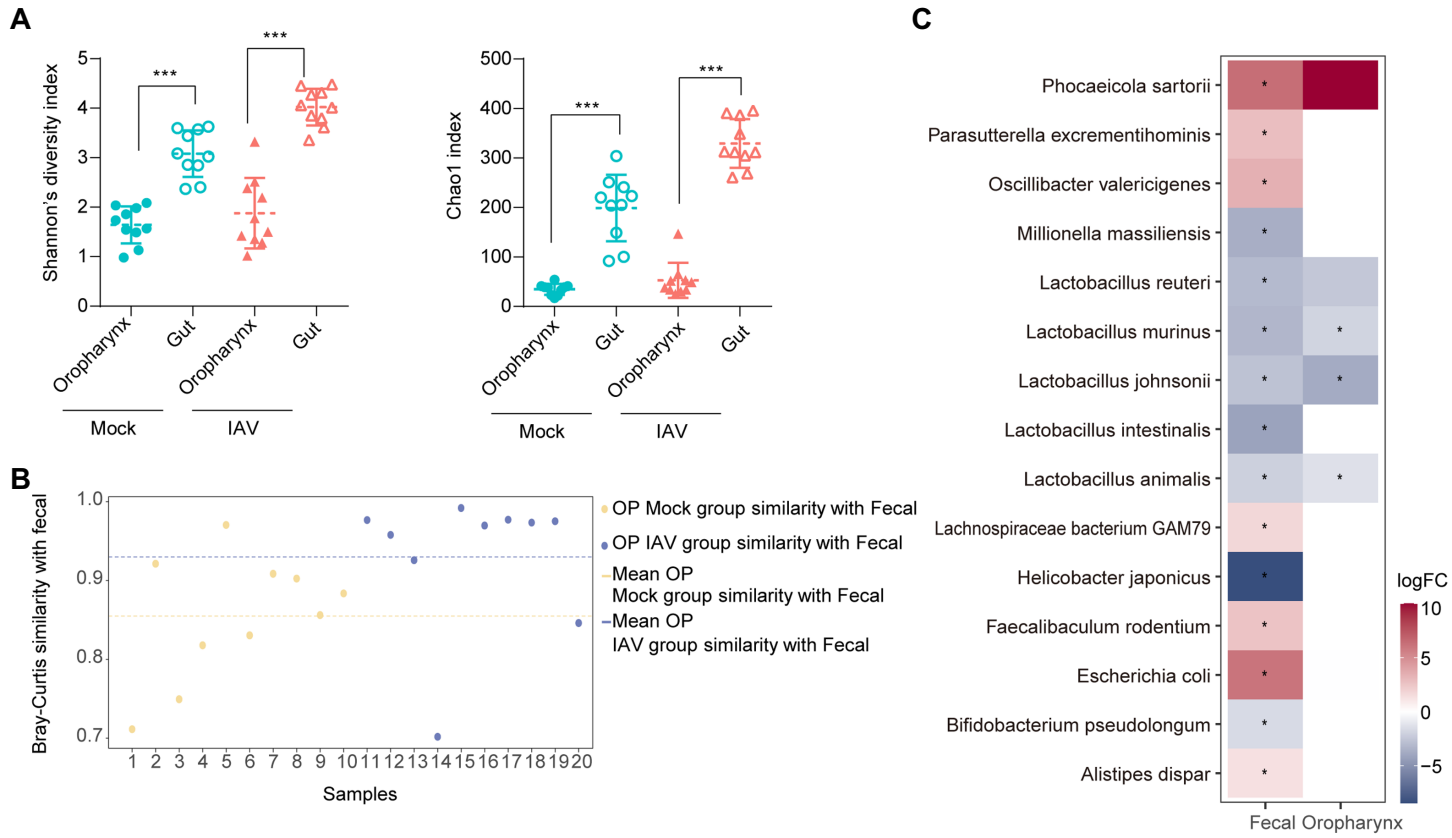
Comparison of oropharyngeal and gut microbiota during IAV infection **(A)** Comparison of the Shannon diversity and Chao1 indices between gut and oropharyngeal microbiota in IAV and mock groups. **(B)** Similarity analysis between gut and oropharyngeal microbiota in IAV and mock groups. **(C)** Distribution of gut and oropharyngeal differential species between mock and IAV groups screened by Lefse analysis. Statistical analyses of Shannon index, Chao1 index and bacterial taxa abundance were conducted by using the Mann–Whitney test. *p < 0.05, ** *p* < 0.01, ***p < 0.001.

IAV infection significantly increased the diversity (Shannon diversity index) and richness (Chao1 index) of gut microbiota (Figure 5A). Beta diversity showed that IAV infection strongly influenced the gut microbiota, as results of the mock and IAV groups clustered away from each other (Figure 5B; Supplementary Figure S1D). *Firmicutes* and *Bacteroidetes* were the dominant gut bacteria in both the IAV and mock groups. However, IAV infection resulted in an increase in the relative abundance of *Bacteroidetes* and a decrease in the relative abundance of *Firmicutes* in gut. At the genus level, IAV infection caused a decrease in the relative abundance of *Lactobacillus* and an increase in the relative abundance of *Muribaculum and Parasutterella* (Figure 5C). LEfSe analysis to identify species-level bacteria associated with IAV infection identified 7 species that were differentially abundant between the mock and IAV groups. *Lactobacillus intestinalis*, *Lactobacillus reuteri*, *L. animalis*, *L. johnsonii*, *Helicobacter japonicus* and *L. murinus* were enriched in the mock group, while *Parasutterella excrementihominis* was significantly more abundant in IAV group (Figure 5D). The metabolic function prediction of microbiota showed that IAV infection decreased D-glutamine and D-glutamate metabolism in gut (Figure 5E). Considering the habitual coprophagy of mice, we analyzed the correlation between gut microbiota and oropharyngeal microbiota in mock and IAV groups and found that the diversity and richness of the gut microbiota were significantly higher than those of the oropharynx in both the IAV and mock groups (Figure 6A). In addition, the oropharyngeal microbiota was more similar to the gut microbiota in mock group than in the IAV group (Figure 6B). We observed the same changes in the relative abundance of some species in the gut and oropharynx after IAV infection. For example, the relative abundance of *L. murinus*, *L. reuteri*, *L. animalis* and *L. johnsonii* were decreased, while the relative abundance of *Phocaeicola sartorii* increased in both gut and oropharynx in IAV group (Figure 6C).

## Discussion

IAV infection can alter respiratory tract and gut microbiota (Lv et al., 2021; Rattanaburi et al., 2022). As murine models are the most commonly used *in vivo* systems in influenza research, many experiments have described long-or short-term dysbiosis in IAV-infected mice (Yildiz et al., 2018; Sencio et al., 2021). However, most studies have focused on a single mucosal surface, and have rarely studied the association of microbiota among multiple locations systematically. Therefore, we compared the respiratory and gut microbiota of normal and IAV-infected mice using full-length 16srRNA gene sequencing and analyzed the correlation of microbiota among different anatomic sites in normal and infected mice.

As expected, we successfully established a murine IAV infection model. Previous murine studies have reported that influenza infection decreases body weight and increases lung index (An et al., 2018; Ling et al., 2020). We observed the same results in our experiments. In addition to the respiratory tract, we also detected IAV in the feces of the IAV-infected group. Concordant with our results, virus was also identified in fecal samples from IAV/IBV-infected patients (Hirose et al., 2016). Our histopathologic findings of robust leukocytic infiltration and edema in IAV-infected lung tissue were also observed in a previous study and were associated with protease-activated receptor 4 (Kim et al., 2021).

The respiratory tract is complex, and each segment has its own unique microbiota, which respond similarly or differently to IAV infection. In this study, species diversity and richness of oropharyngeal, nasopharyngeal and pulmonary microbiota in IAV-infected mice were similar to those of normal mice. Consonant with our findings, several studies have shown no significant changes in microbial diversity and richness in the URT and LRT of IAV-infected mice (Planet et al., 2016; Yildiz et al., 2018). However, the composition of the microbiota of the different sites responded differently to IAV infection.

The reduction of relative abundance of *L. murinus* at all three respiratory tract sites may have altered host immune response and may have also reduced colonization resistance, as evidenced by the increased relative abundance of oropharyngeal and nasopharyngeal *P. fluorescens. L. murinus* belong to *Lactobacillus* genus which induces Th17 and RORγt+ regulatory T cells and reduces pulmonary inflammation in tuberculosis (Bernard-Raichon et al., 2021). Moreover, *L. murinus* provides a barrier against pneumococcal colonization in a respiratory dysbiosis model (Yildiz et al., 2020). *P. fluorescens* belongs to *Pseudomonadales*, which typically subsist at low levels in the indigenous microbiota of various body sites, but are related to cystic fibrosis, chronic airway diseases, asthma and non-cystic fibrosis-related bronchiectasis (Scales et al., 2014). The relative abundance of *Pseudomonadales* also increased in the URT of IAV-infected patients (Kaul et al., 2020).

Interestingly, we found that the relative abundance of *S. danieliae* was increased in the URT and decreased in the LRT. *S. danieliae* is a major component of the URT microbiome of healthy mice and is involved in the establishment of oral microbiota (Joseph et al., 2021). However, oral administration of *Staphylococcus aureus* and *S. danieliae* aggravated experimental psoriasis in a murine model (Okada et al., 2020). There have been few other reports of *S. danieliae* to date. Our research showed that the lung microbiota was more like that of the nasopharynx than the oropharynx, which suggests that the nasopharynx may serve as the primary reservoir of lung microbiota in mice. In contrast, the oropharynx is the origin of lung microbiota in human adults (Bassis et al., 2015). The nasopharyngeal microbiota is most similar to the lung microbiota in healthy cattle (McMullen et al., 2020). Moreover, IAV infection increased the similarity between nasopharyngeal and lung microbiota in this study. This may have been related to host innate and adaptive immune responses to IAV (Liong et al., 2020; Wang et al., 2021).

IAV infection can decrease the species diversity of gut microbiota in H7N9-infected humans (Qin et al., 2015). However, our results showed that IAV infection increased intestinal species diversity in mice. This could be due to different viral strains, host responses, or sample collection methods. The relative abundance of *Lactobacillus* spp. (*L. intestinalis*, *L. reuteri*, *L. animalis*, *L. johnsonii* and *L. murinus*) was decreased in IAV-infected mice. Considering the absolute abundance can be estimated from the sequencing counts (Yang et al., 2022), we also evaluated the absolute abundance of *Lactobacillus* in the genus and species levels between the IAV and Mock groups, and found that change of *Lactobacillus* was consistent in the absolute and relative abundance (Supplementary Table S2). In general, the decreased abundance of Lactobacillus species may be an important phenomenon of IAV infection. *Lactobacillus* plays an important role in anti-viral immunity. For example, a previous study showed that *Lactobacillus johnsonii* supplementation attenuates respiratory viral infection *via* immune cell modulation (Fonseca et al., 2017). Moreover, the relative abundance of *L. murinus* was not only decreased in the gut, but also in all three segments of the respiratory tract (oropharynx, nasopharynx, lungs). Reduced relative abundance of *L. murinus* at all anatomic sites may have reduced colonization resistance. *L. murinus* maintained intestinal immune homeostasis and mediated anti-inflammatory effects in murine models (Tang et al., 2015; Pan et al., 2018). Probiotic administration of *L. murinus* prevented salt-sensitive hypertension by modulating TH17 cells in mice (Wilck et al., 2017). *Parasutterella excrementihominis* was significantly more abundant in gut microbiota in IAV infected mice, and was found in a higher relative abundance in older adults (Fart et al., 2020). Increased relative abundance of *P. excrementihominis* has been associated with fatty liver disease, chronic bowel inflammation and irritable bowel syndrome (Blasco-Baque et al., 2017; Chen et al., 2018). In addition, IAV infection reduced the similarity of oropharyngeal and gut microbiota. The correlation between murine oropharyngeal and intestinal microbiota may be due to coprophagy (Bogatyrev et al., 2020). Because IAV infection reduces murine alimentation (Bartley et al., 2017), decreased fecal feeding may have lowered the similarity between oropharyngeal and gut microbiota in our infected mice. The murine behavior of coprophagy and subsequent respiratory colonization by enteric microflora brings into question the utility of murine models of post-viral bacterial superinfections.

## Conclusion

We characterized the oropharyngeal, nasopharyngeal, lung and gut microbiota and compared the microbiota structure of different mucosal surfaces in normal and IAV-infected mice. In addition, we determined that the nasopharynx is the primary reservoir of lung microbiota in healthy mice. IAV infection increased the similarity between lung and nasopharyngeal microbiota. However, IAV infection reduced the similarity between oropharyngeal and gut microbiota. The relative abundance of *L. murinus* may serve as a biomarker of IAV infection because it was reduced in all locations.

## Data availability statement

The data presented in the study are deposited in the NCBI Bio-project repository (https://www.ncbi.nlm.nih.gov/bioproject/), accession number PRJNA910300.

## Ethics statement

The animal study was reviewed and approved by Animal Care and Use Committee of Chinese PLA Center for Disease Control and Prevention.

## Author contributions

PenL and HS conceived and designed the experiments. QC, KW, LJ, YL, and JL performed the experiments. ML, HG, BZ, PeiL, and LY analyzed the full length 16 s rRNA gene sequencing data. QC and PenL wrote the manuscript. All authors contributed to the article and approved the submitted version.

## Funding

This study was supported by National Key Research and Development Program of China (2021YFC2301000), the National Science and Technology Major Project (2018ZX10201001 and 2018ZX10305410).

## Conflict of interest

ML, BZ, and HG were employed by the companies Jiangsu Simcere Diagnostics Co., Ltd. and Nanjing Simcere Medical Laboratory Science Co., Ltd.

The remaining authors declare that the research was conducted in the absence of any commercial or financial relationships that could be construed as a potential conflict of interest.

## Publisher’s note

All claims expressed in this article are solely those of the authors and do not necessarily represent those of their affiliated organizations, or those of the publisher, the editors and the reviewers. Any product that may be evaluated in this article, or claim that may be made by its manufacturer, is not guaranteed or endorsed by the publisher.
