# Supplementary material for: Topography of respiratory tract and gut microbiota in mice with influenza A virus infection
Source: Front Microbiol. 2023 Feb 22;14:1129690. doi: 10.3389/fmicb.2023.1129690 (PMC9992211; doi:10.3389/fmicb.2023.1129690)
Supplement: Supplementary file 3 [file Data_Sheet_1.PDF]

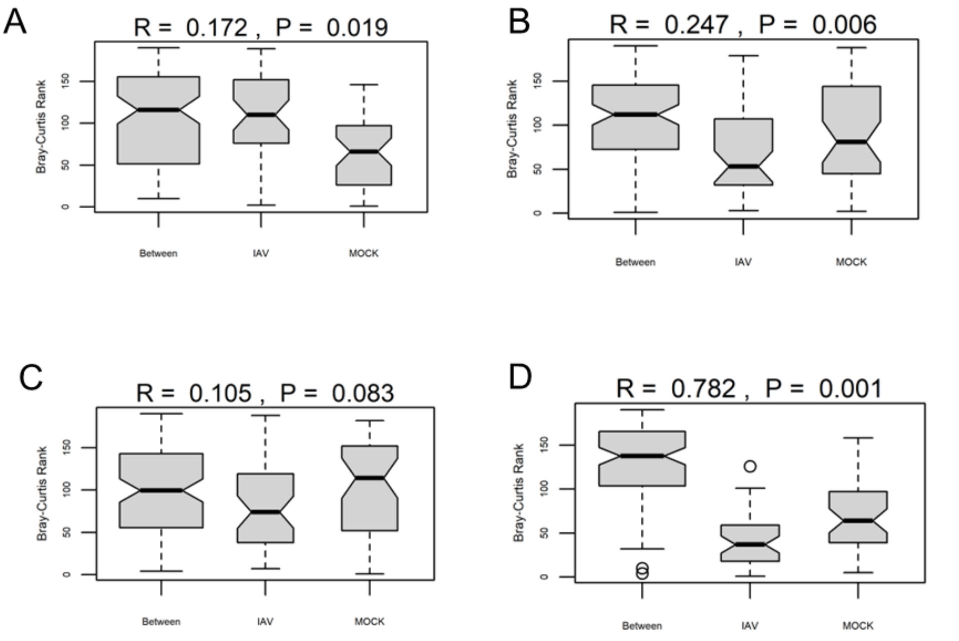

FIG 1: Bray-Curtis rank of microbiota between mock and IAV group. (A) Bray-Curtis rank of oropharyngeal microbiota between mock and IAV group. (B) Bray-Curtis rank of nasopharyngeal microbiota between mock and IAV group. (C) Bray-Curtis rank of lung microbiota between mock and IAV group. (B) Bray-Curtis rank of gut microbiota between mock and IAV group. Bray-Curtis rank was analyzed by the ANOSIM.
